# Lifestyle Habits and Adherence to Cancer Screening Programs Among Italian Teachers: A Cross-Sectional Study

**DOI:** 10.3390/healthcare13233080

**Published:** 2025-11-26

**Authors:** Giovanna Paduano, Silvia Angelillo, Vincenza Sansone, Concetta Paola Pelullo, Francesco Napolitano, Gabriella Di Giuseppe

**Affiliations:** 1 Department of Experimental Medicine, University of Campania “Luigi Vanvitelli”, Via Luciano Armanni 5, 80138 Naples, Italy; giovanna.paduano@unicampania.it (G.P.); silvia.angelillo@studenti.unicampania.it (S.A.); vincenza.sansone@unicampania.it (V.S.); gabriella.digiuseppe@unicampania.it (G.D.G.); 2 Department of Medical, Human Movement and Well-Being Sciences, University of Naples “Parthenope”, 80133 Naples, Italy; concettapaola.pelullo@uniparthenope.it

**Keywords:** behaviors, cancer screening, Italy, lifestyle habits, physical activity, survey, teachers

## Abstract

**Objectives:** This study aims to evaluate teachers’ lifestyle habits and to investigate their knowledge and behaviors related to cancer screening. **Methods:** This cross-sectional survey was performed among teachers randomly selected from schools located in the Campania region, Italy. **Results:** Only 17% of the teachers were current smokers, while 72.1% consumed alcohol. Female teachers, those who were married/cohabitant, and those who discussed with students about alcohol consumption were more likely to have never smoked or drunk alcohol. Female and older teachers, those with a university or a master/PhD degree, and those who had a moderate/high level of physical activity (PA) were more likely to sufficiently consume fruits and vegetables. Only 20.9% of teachers had a moderate/high level of PA. Those who had at least one child, who taught humanistic and support disciplines, and who needed additional information on healthy lifestyle habits were less likely to have a moderate/high level of PA. Among participants, 42.3% had ever undergone mammography for screening and 37.5% a Pap test and a fecal occult blood test. **Conclusions:** This survey describes a worrying prevalence of unhealthy behaviors and low adherence to screening programs among Italian teachers, suggesting the need for education and screening campaigns to improve preventive strategies in this population.

## 1. Introduction

It is well known that unhealthy habits are relevant risk factors for non-communicable diseases and cancers, and there is a large body of evidence regarding the effectiveness of public health education interventions to promote healthy behaviors in order to reduce the burden of these conditions [1,2,3]. However, it is essential that individuals receive the appropriate information about risk factors and have access to prevention measures, such as screening programs, provided by health authorities in order to enhance their ability to make responsible choices for their own health [4,5].

The evaluation of teachers’ awareness and behaviors regarding healthy habits and prevention measures can provide important information since teachers’ lifestyles could have a relevant influence on the wellbeing of their students given that, within the framework of social learning theory, students learn through observation and imitation of their behaviors [6]. Indeed, previous investigations have shown that teachers who adopt healthy habits, such as proper nutrition and regular physical activity, positively influence students’ behaviors and promote healthy practices during classroom activity [7,8,9,10]. Moreover, teachers may play a key role in health education during their stay in schools by spreading correct knowledge about risk factors for non-communicable diseases and cancers, and being a virtuous example through their appropriate attitudes and behaviors [11,12]. School-based health education with teachers’ involvement has been shown to improve adolescents’ knowledge, attitudes, and behaviors regarding cancer prevention, which can extend to their families [13,14,15]. Finally, teachers could collaborate with health authorities and healthcare workers to promote healthy lifestyles within the population and, above all, to encourage the maintenance of wellbeing throughout childhood, adolescence, and adulthood [16,17,18].

To our knowledge, only a few previous studies have investigated teachers’ lifestyles, focusing mainly on smoking habits, diet, and work-related stress [19,20,21]. Therefore, it is interesting to investigate more comprehensively their health-related behaviors, in order to gather useful information to better plan and optimize health promotion interventions and preventive strategies. Indeed, this professional group is of particular concern in Italy since they play a fundamental role in children and adolescents’ education due to the number of hours they spend in school, but they often have precarious jobs. They play an important role in influencing public health, since many educational programs developed in Italy include the participation of teachers as an active part.

This investigation, therefore, has three main research questions:

1. What are the lifestyle habits of teachers (including smoking, alcohol consumption, dietary habits, and physical activity)?
2. What is the level of teachers’ knowledge and behaviors regarding cancer screening?
3. Which factors influence teachers’ lifestyle habits, knowledge, and behaviors related to cancer screening?

## 2. Materials and Methods

### 2.1. Study Design and Setting

This cross-sectional survey was conducted as part of a broader project investigating lifestyles and healthy behaviors among different groups of the Italian population [22,23,24]. The study was conducted from February to April 2024 among a randomly selected sample of teachers working in kindergarten and primary, middle, and high schools located in Naples and Avellino, two cities in the Campania region, Italy.

The sample was selected through a one-stage cluster sampling method. The teacher population was divided into clusters consisting of all the public schools in the Campania region. Then, from the list of all eligible schools, the research team selected, through a simple random procedure, a sample of these clusters composed of three high schools and three Comprehensive Institutes (kindergarten and primary and middle schools). Subsequently, all the teachers within these selected schools were invited to participate in the study. The minimum total sample size was estimated at 496 teachers, assuming a prevalence of 70% [25] of teachers who had had an oncological screening test and considering a 95% confidence interval, an alpha error of 5%, and an expected response rate of 65% [26].

The deans of the selected schools received a letter with an invitation to take part in the survey, describing the aims and methodology of the study. Then, the teachers received an email from the school dean including a link to the anonymous online questionnaire which was on the Google Drive platform and administered in Italian. The questionnaire included a letter describing that privacy and anonymity were stringently guaranteed. Participation in the survey was voluntary and no money or gifts for their participation in the study were provided. Furthermore, it was specified that completing and sending back the questionnaire would have been considered implicit consent to participation. Two reminders were sent after two and four weeks from the beginning of the study, in order to improve the response rate.

A pilot study was conducted on 50 teachers to assess the validity and exhaustiveness of the data collection instrument. Since no changes were made to the survey tool, the results of the pilot study were included in the final sample. The study protocol and the questionnaire were approved by the Ethics Committee of the Teaching Hospital of the University of Campania, “Luigi Vanvitelli” (approval number: 0018199/i 01.07.2024).

### 2.2. Survey Instruments

The self-administered questionnaire (Supplementary File S1) aimed to collect data in the following three sections: (1) sociodemographic, professional, and anamnestic characteristics of teachers such as age, gender, marital status, educational level, number and characteristics of cohabitants, number of children, presence of at least one non-communicable disease, weight and height to obtain the Body Mass Index (BMI: underweight: <18.5; healthy weight: 18.5–24.9; overweight: 25–29.9; obesity: ≥30), type of school, and teaching discipline; (2) lifestyle behaviors (smoking status, alcohol intake, physical activity, daily consumption of fruits and vegetables, daily portions of dietary protein sources, frequency of consumption of snacks and sweets, and assessment of sleep quality), sources of information about lifestyle behaviors, and the need for additional information; and (3) knowledge and participation in cancer screening programs through mammography, the Papanicolaou test (Pap test), the human papillomavirus DNA test (HPV DNA test), and the fecal occult blood test (FOBT), sources of information, and the need for additional information about the screening programs. In all sections, data were collected using closed-ended questions with multiple-choice answers.

Teachers eligible for cancer screening programs were women aged 45–69 years for mammography, women aged 25–64 years for the Pap test, women aged 30–64 years for the HPV-DNA test, and men and women aged 50–69 years for the FOBT.

The International Physical Activity Questionnaire—Short Form (IPAQ-SF) was used to measure the physical activity level. It consists of seven items evaluating total energy expenditure per week by considering the number of days and minutes spent on vigorous physical activity (8 METs), moderate physical activity (4 METs), and walking (3.3 METs for an intense pace, 3 METs for moderate intensity, and 2.5 METs for a slow pace). The IPAQ total value is stated in MET-min/week, and three categories were recognized based on total METs: inactive (< 700 METs), moderately active (701–2519), and highly active (> 2520) [27,28].

Sleep quality was measured using the 19-item Pittsburgh Sleep Quality Index (PSQI) [29,30]. The PSQI is a self-administered questionnaire that estimates the quality of sleep with questions about sleep over a one-month time interval. The scale is composed of the following 7 components: subjective sleep quality, sleep latency, sleep duration, habitual sleep efficiency, sleep disturbances, use of sleep medications, and daytime dysfunctions. The score of the PSQI has a range between 0 and 21, with a higher score indicating a lower quality of sleep. A score  ≥5 indicates poor sleep, while <5 indicates good sleep.

Before starting the investigation, a pilot study was conducted on 50 teachers to ensure the correct interpretation, clarity, and feasibility of the questionnaire. No changes were made to the survey instrument, and the results were included in the final sample.

### 2.3. Statistical Analysis

All data were anonymized and handled on a password-protected university server accessible only to the research team. Stata statistical software version 17 was used to conduct all statistical analyses [31]. Descriptive statistics was carried out to present frequencies and proportions for categorical variables, and means with standard deviations for continuous variables. Cases with missing information on key variables were listwise deleted from the relevant analyses. Before building the multivariable models, each independent variable was examined in univariate analysis to test associations with the different outcomes of interest using the chi-square test for categorical variables and the Student *t*-test for continuous variables. Then, in order to ensure that potentially relevant variables were included in the models, all variables with a *p* value ≤ 0.25 at univariate analysis were included in the following seven stepwise multivariate logistic regression models according to the Hosmer and Lemeshow model building strategy [32]: having never smoked and never drunk alcohol (no = 0; yes = 1) (Model 1); eating at least 5 daily portions of fruits and vegetables (no = 0; yes = 1) (Model 2); having a moderate/high level of physical activity (no = 0; yes = 1) (Model 3); having discussed at least one topic among nutrition, physical activity, cigarette smoking, and alcohol consumption with students at school (no = 0; yes = 1) (Model 4); having had a mammography for breast cancer screening (no = 0; yes = 1) (Model 5); having had a Pap smear for cervical cancer screening (no = 0; yes = 1) (Model 6); and having had a FOBT for colorectal cancer screening (no = 0; yes = 1) (Model 7). The independent variables included in the different final models are shown in a Supplementary File (Supplementary File S2).

Adjusted odds ratios (ORs) and 95% confidence intervals (CIs) were calculated. All reported *p* values were two-tailed, and a value ≤0.05 was considered statistically significant.

## 3. Results

### 3.1. Sociodemographic, Professional, and Anamnestic Characteristics of Teachers

Out of 498 teachers invited, 323 completed the questionnaire, with a response rate of 64.8%. Table 1 shows the sociodemographic, professional, and anamnestic characteristics of the participants.

### 3.2. Lifestyle Behaviors

Table 2 reports teachers’ lifestyle behaviors and sources of information. It is interesting that the results describe how teachers’ lifestyle behaviors are often unhealthy. However, more than half of the participants (57.9%) had never smoked, whereas only 17% were current smokers, with a mean number of 11.6 cigarettes per day. A large majority of smokers used traditional tobacco products (81.8%), while 16.4% used heated tobacco products. Approximately two-thirds of participants (62.5%) declared being exposed to passive smoking at least one day during the previous week. Regarding alcohol consumption, 72.1% of participants consumed alcohol, 20.7% of them once a month or less, 30% two to four times per month, 14.6% two or three times per week, and 6.8% four or more times per week. Among drinkers, almost all (95.4%) reported drinking at most 1–2 drinks on the days they drink, 3.7% 3–4 drinks, and 0.9% 5–6 drinks.

The results of the multivariate logistic regression model showed that female teachers (OR = 2.48; 95% CI = 1.02–6.06; *p* = 0.046), those who were married/cohabitant (OR = 2.43; 95% CI = 1.17–5.04; *p* = 0.017), and those who talked with their students about alcohol consumption (OR = 2.35; 95% CI = 1.07–5.12; *p* = 0.033) were significantly more likely to have never smoked and never consumed alcohol (Model 1 in Table 3).

Less than half of the sample (41.5%) reported healthy dietary habits with regard to the consumption of at least five daily portions of fruits and vegetables, 25.5% consumed two weekly portions of dietary protein sources, and 27.6% reported rare consumption of snacks and sweets. Female (OR = 2.24; 95% CI = 1.11–4.49; *p* = 0.024) and older teachers (OR = 1.03; 95% CI = 1.01–1.06; *p* = 0.024), those who had a university (OR = 4.25; 95% CI = 1.36–13.29; *p* = 0.013) or a postgraduate degree (OR = 4.23; 95% CI = 1.14–15.74; *p* = 0.031), those who were overweight (OR = 2.87; 95% CI = 1.02–8.09; *p* = 0.046), and those who engaged in physical activity (OR = 2.14; 95% CI = 1.31–3.55; *p* = 0.003) were more likely to eat at least five daily portions of fruits and vegetables (Model 2 in Table 3).

Only one in five teachers (20.9%) reported a moderate/high level of physical activity, and 45.9% were inactive according to the IPAQ score. The multivariate logistic regression analysis indicates that education and information play a crucial role in the development of healthy dietary and physical activity behaviors and it showed that those eating at least five daily portions of fruits and vegetables (OR = 2.38; 95% CI = 1.43–3.95; *p* = 0.001) were more likely to have a moderate/high level of physical activity, while those who had at least one child (OR = 0.52; 95% CI = 0.29–0.93; *p* = 0.027), who taught in the humanistic (OR = 0.53; 95% CI = 0.29–0.94; *p* = 0.029) and support disciplines (OR = 0.33; 95% CI = 0.16–0.71; *p* = 0.004), and who needed additional information on healthy lifestyle habits (OR = 0.55; 95% CI = 0.34–0.91; *p* = 0.019) were less likely to perform this healthy behavior (Model 3 in Table 3).

Regarding the evaluation of sleep quality, the mean value of the PSQI score among the participants was 6.2  ±  3.1, and, in particular, two-thirds of men (65.5%) and women (67.9%) had poor sleep with a PSQI score of ≥5.

Almost all respondents reported having had at least one specialist visit in the last year, and the most frequently consulted specialists were rheumatologists (90.1%), dentists (72.1%), ophthalmologists (52.3%), dermatologists (35%), and gynecologists (35%).

More than two-thirds of participants (72.8%) had received information about lifestyle habits, and the most common sources of information were the Internet and social media (54%), followed by physicians (44.9%), friends and family (22.4%), school (22.4%), and TV and journals (5.9%). Moreover, 41.8% of teachers reported the need for additional information on healthy lifestyle habits, suggesting the need for public health programs directed at this population.

Regarding the topics discussed with their students at school, the results underlined that schools represent a fundamental setting for the spread of information on healthy behaviors. Indeed, teachers had declared talking with them about nutrition (74.5%), physical activity (57.4%), cigarette smoking (45.9%), and alcohol consumption (41.9%). Moreover, 51% of teachers discussed at least one topic among nutrition, physical activity, cigarette smoking, and alcohol consumption with students at school. Male teachers (OR = 0.39; 95% CI = 0.19–0.78; *p* = 0.007) and those who had a university degree (OR = 4.55; 95% CI = 1.64–12.64; *p* = 0.004) were more likely to have discussed at least one topic with students at school, while those who taught humanistic (OR = 0.43; 95% CI = 0.21–0.89; *p* = 0.022), artistic (OR = 0.44; 95% CI = 0.22–0.88; *p* = 0.02), and support disciplines (OR = 0.38; 95% CI = 0.16–0.91; *p* = 0.03), compared with those who taught science, were less likely to have discussed at least one topic (Model 4 in Table 4).

### 3.3. Knowledge and Uptake of Cancer Screening Among Eligible Teachers

Teachers’ knowledge regarding cancer screening programs is reported in Table 5, and the relative participation of eligible teachers in Table 6.

A large majority of participating women (84.2%) correctly reported that mammography is free of charge for those aged 45–69 years, whereas more than half (53.8%) were unaware that the screening interval was two years.

The results on the teachers’ behaviors regarding screening programs showed that the adherence to screening programs is, overall, not sufficient, and public health interventions are needed to improve these outcomes. Indeed, 188 (91.3%) eligible teachers had ever undergone mammography, 50% as a diagnostic exam and 50% for screening purposes. Among those who had undergone mammography for screening, 44.3% participated in an opportunistic procedure, and 55.7% in an organized screening program. Adherence to the screening interval of two years was reported by 90.6% of the women. The main reported reasons for not having undergone mammography were lack of time (43.8%) and not having received a recommendation by physicians (18.8%). The multivariate logistic regression analysis showed that those who had received information about screening programs (OR = 3.56; 95% CI = 1.39–9.11; *p* = 0.008) were significantly more likely to have undergone mammography, whereas those who had a university degree (OR = 0.32; 95% CI = 0.11–0.97; *p* = 0.046) were significantly less likely to have a mammogram for breast cancer screening (Model 5 in Table 7), underscoring, again, the important role of information in developing healthy preventive behaviors.

More than half of the participating women (57.9%) correctly reported that the Pap test is free of charge for women aged 24–64 years, whereas only 23.5% were aware that the screening interval was three years. Overall, 231 (95.1%) eligible teachers had ever undergone a Pap test and 37.5% of them did so for screening purposes; moreover, 31.4% of those who underwent a Pap test for screening only participated in an organized program. The main reported reasons for non-participation in cervical cancer screening were not having been informed by physicians (50%) and lack of time (37.5%). The results of the multivariate model highlighted that those who were married/cohabitant (OR = 2.59; 95% CI = 1.22–5.47; *p* = 0.013), those who had a family member with at least one non-communicable disease (OR = 2.11; 95% CI = 1.13–4.33; *p* = 0.021), and those who knew that the test is free of charge were more likely to participate in a screening program, whereas this behavior was less likely among those with a university degree (OR = 0.31; 95% CI = 0.11–0.87; *p* = 0.026) (Model 6 in Table 7).

About half of the participating women (51.2%) correctly reported that the HPV-DNA test is free of charge for women aged 30–64 years. Overall, only 32.1% of the teachers had ever undergone an HPV-DNA test, and 45.7% of them for screening purposes; moreover, 53.1% of those who underwent an HPV-DNA test for screening participated in an organized program. The main reported reasons for not having undergone the HPV-DNA test were not having been informed by physicians (88.7%) and lack of time (13.5%).

More than two-thirds of the sample (69.1%) correctly reported that the FOBT is free of charge for those aged 50–69 years, and 39.9% correctly reported that the screening interval was two years. Overall, 78 (37.5%) participants had ever undergone a FOBT, and 59.7% of them for screening purposes. Among those who underwent a FOBT for colorectal cancer screening, 79.1% participated in organized program. The main reported reasons for not having been screened for colorectal cancer were not having been informed by physicians (73.8%) and lack of time (29.6%). The results of the multivariate logistic regression analysis showed that the older teachers (OR = 1.09; 95% CI = 1.02–1.18; *p* = 0.014) were significantly more likely to have undergone a FOBT (Model 7 in Table 7).

The large majority of teachers (83.6%) had received information about screening programs. Physicians (63.1%) and the TV/journals/media (39.9%) were indicated as the two main sources of information, and 62.2% of teachers acknowledged that they need additional information on cancer screening programs.

## 4. Discussion

To the best of our knowledge, this is the first study conducted in Italy that has investigated lifestyle habits and cancer screening behaviors among teachers. The results depict non-optimal adherence to screening programs and a high prevalence of unhealthy behaviors among the selected sample. In particular, regarding the last point, the number of current smokers among teachers was higher than that reported in China [33], but lower than those reported among the general population in Italy [34] and for teachers in Chile [35]. In addition, regarding alcohol consumption, the presented value is higher than that reported in China, where the majority of teachers declared themselves to be noncurrent drinkers [33], and consistent with the results reported in Japan [36]. Differences may be due to the different health policies and prevention campaigns against unhealthy behaviors developed in these countries, suggesting the opportunity for future campaigns against smoking and alcohol consumption to promote healthy behaviors.

Regarding the low consumption of daily portions of fruits and vegetables described in this sample, it is interesting to note that it has been reported that teachers with lower fruit intake per day are likely to have higher scores for emotional exhaustion and depersonalization [37]. Therefore, it is important to better investigate this aspect since emotional difficulties are generally extensive among teachers [38], possibly due to burnout and stress. This would have an impact on professional performance, highlighting the need for interventions to improve teachers’ emotion regulation.

Another aspect on which to focus is the high level of physical inactivity, similar to data reported elsewhere [39]. Teaching is a sedentary job; therefore, it is important to promote healthy behaviors regarding eating and physical activity to avoid the spread of unhealthy behaviors and the consequential development of non-communicable diseases.

Regarding the evaluation of poor sleep quality, the reported data are worse than those reported in a similar study [40]. These data need to be better explored since it has been described in the literature on teachers that more emotional exhaustion and higher recovery needs were associated with less sleep per night during the week [37].

The need for additional information on healthy lifestyle habits, and the findings that not all teachers discussed with their students regarding these issues, might suggest that teachers are not prepared to teach about healthy behaviors and they do not perceive themselves to have a role in changing students’ unhealthy lifestyles [41]. For this reason, it could be useful to plan interventions by healthcare professionals focused on the development of skills for the promotion of healthy lifestyles among teachers and students.

Interestingly, the results of the multivariate logistic regression model on the level of physical activity confirmed that education has an important role in healthy behaviors and is a fundamental social determinant of health [42]. This is in line with another result describing that more educated teachers were more likely to eat at least five daily portions of fruits and vegetables. These data are confirmed by the literature, depicting that a high educational level is linked to greater consumption of fruits and vegetables [43]. Moreover, teachers who discussed with their students about alcohol consumption were significantly more likely to have never smoked or drunk alcohol. This can be explained because high health literacy was associated with healthy behaviors and may increase confidence to talk to others about these themes [44], confirming again the importance of future health education strategies for this particular population.

Moreover, the results of the multivariate logistic regression model showed that female teachers were significantly more likely to have healthy behaviors, and this is in line with the previous literature [45,46,47]. Understanding this gender specificity may be useful for targeted public health interventions focused on men. Also, marital status appears to have a role in healthy behaviors. Previous studies have also described that marriage encourages healthy behaviors and that these benefits continue later in life, with better health outcomes [48,49]. Moreover, this study showed that having a moderate/high level of physical activity and eating at least five daily portions of fruits and vegetables are correlated, as reported elsewhere [50], but this result is not in line with the previous literature describing that teachers who reported frequent sedentary breaks were more likely to consume fruits and vegetables [39]. Despite the disagreement, these behaviors appear to have important inter-relationships that require further investigation to understand optimal interventions and conditions for their promotion.

Regarding the uptake in oncological screening, the adherence rates to all recommended screening programs (breast, colorectal, and cervical cancers) were not optimal and are lower than those observed in the general population in Italy [51].

Similar findings have been reported for colorectal cancer in two previous investigations conducted in similar geographic areas [52,53,54]. Instead, higher adherence to cancer screening guidelines compared to those of the present study was observed among adults in Korea and the US [55,56,57,58] and in a survey conducted in England, where self-reported screening uptake was higher [59], suggesting that the national approach to screening program promotion could have a role in uptake. Moreover, it is interesting to note that in our study the main reported reasons for not participating in screening programs were not having been informed by physicians and lack of time, although the most reported source of information on screening was physicians. Several factors have been reported to affect whether patients undergo screening, and similar reasons have been confirmed by previous investigations [53,60,61,62]. Therefore, it is clear that there is room for improving the effectiveness of current screening programs for breast, cervical, and colorectal cancers. Many studies in the literature have evaluated interventions to increase screening adherence [63,64,65,66,67,68,69] whereas, considering the results of this study, the strategies should focus on support by healthcare workers to increase awareness of screening and encourage demand for it, and to facilitate access to screening tests in healthcare facilities by bringing screening tests closer to users. This objective can be achieved through multi-pronged approaches involving patients, healthcare workers, and healthcare policy makers, and assuring, for example, greater flexibility in opening hours of facilities; and through the diversification of screening settings, such as pharmacies, general practitioners, outpatient specialists, and mobile screening units. In this complex approach, teachers can play a key role in health education and awareness, helping to create a culture of prevention among young people that could lead to increased adherence to screening in adulthood. Indeed, the school represents a strategic setting to promote health, and investment has to be made in teachers’ pre-service training in health promotion, since their role is to tailor learning strategies and activities to the developmental needs of students [70].

A major aim of this investigation was to evaluate the variables that influence screening adherence among the participants. According to the results, Pap test uptake was significantly more likely among those who are married, and this result has previously been observed in several studies [71,72,73,74]. This finding could be explained by the fact that married women have greater support from their partners or family in facing a prevention intervention that could cause anxiety and worry. Another important factor among the sociodemographic characteristics influencing screening uptake was age, with older teachers being more likely to have undergone a FOBT. This finding is in line with those of previous surveys [75,76,77,78]. This association could be explained by the fact that older people have more frequent access to health services, for other non-communicable conditions as well, and could benefit from the recommendation of screening tests by healthcare professionals. For this reason, a screening campaign could be organized in schools to facilitate teachers’ access to preventive programs.

This study has potential methodological limits which must be taken into consideration in interpreting the results. First, no causal inference between the predictors and the different outcomes of interest can be established due to the cross-sectional study design. Second, the teachers’ sampling was carried out only in public schools in two cities in the Campania region, and thus this may limit the representativeness and generalizability of the results to Southern Italy. Third, data were collected using a self-administered questionnaire and participants self-reported information about their habits and cancer screening uptake without confirmation from the medical records, and, therefore, it is possible that there were desirability, self-report, and recall biases. Despite these limitations, this study explores important behaviors regarding a target professional category that plays a crucial role in health education.

## 5. Conclusions

In conclusion, despite the cross-sectional nature of this study and the regional sample limiting its generalizability, the results indicate that teachers need to be better informed regarding healthy behaviors and cancer screening programs. In particular, physicians’ information appears to have a strategic role in teachers’ knowledge and behaviors regarding health. School policies need to invest in professional learning by teachers, including the presence of healthcare staff in the school; in health education strategies; and in using the school setting to involve teachers directly, for example, in screening programs. Teachers’ curricula should expand the possibilities of knowing healthy behaviors to stimulate the teachers to share them in the classroom.

## Figures and Tables

**Table 1:** Sociodemographic, professional, and anamnestic characteristics of the study population.

Characteristics	Total (N. 323)	
*Sociodemographic*	**N**	**%**
**Gender**		
Male	58	18
Female	265	82
**Age group (years)**	51.9 ± 9.2 (26–67) *	
≤50	128	39.6
>50	195	60.4
**Marital status**		
Unmarried/widowed/divorced	83	25.7
Married/cohabitant	240	74.3
**Education level**		
High school	26	8.1
University degree	255	78.9
Postgraduate degree (master, PhD)	42	13
**Partner’s education level ^a^**		
High school	103	42.9
University degree	126	52.5
Postgraduate degree (master, PhD)	11	4.6
**Children**	1.9 ± 0.7 (1–4) *	
No	83	25.7
At least one	240	74.3
**Number of cohabitants**	2.2 ± 1.2 (0–5) *	
*Anamnestic*		
**Non-communicable disease**		
No	231	71.5
At least one ^b^	92	28.5
Endocrinological diseases	24	21.8
Cardiovascular diseases	19	17.3
Diabetes	8	7.3
Respiratory diseases	4	3.6
Oncological diseases	4	3.6
Others (e.g.: ophthalmological, dermatological, rheumatological diseases, etc.)	33	46.4
**Family member with non-communicable disease**		
No	248	76.8
At least one	75	23.2
**Body Mass Index (BMI) ^b^**	24.7 ± 3.9 (17.7–38.8) *	
Underweight	3	0.9
Healthy weight	187	58.1
Overweight	100	31.1
Obesity	32	9.9
*Professional*		
**Type of school**		
Kindergarten	14	4.3
Primary school	31	9.6
Middle school	132	40.9
High school	146	45.2
**Disciplines**		
Humanistic	89	27.6
Science	67	20.7
Artistic	114	35.3
Support	53	16.4

* Mean ± standard deviation (range). ^a^ Only among those who had a partner. ^b^ Numbers for each item may not add up to the total number of the study population due to missing values.

**Table 2:** Teachers’ lifestyle behaviors and sources of information.

Characteristics	Total (N: 323)	
*Lifestyle behaviors*	**N**	**%**
**Smoking status**		
Never smoked	187	57.9
Former smokers	81	25.1
Current smokers	55	17
**Number of cigarettes ^a,b^**	11.6 ± 6.6 (1–25) *	
**Alcohol consumption (Yes)**	233	72.1
**Physical activity status (IPAQ score) ^c^**		
Inactive	125	45.9
Minimally active	90	33.1
Active/very active	57	20.9
**PSQI score**	6.2 ± 3.1 (0–16) *	
Poor sleep	218	67.5
Good sleep	105	32.5
**At least 5 daily portions of fruits and vegetables (Yes)**	134	41.5
**Two daily portions of dietary protein (Yes)**	81	25.1
**Rare consumption of snacks and sweets (Yes)**	89	27.5
*Sources of information*		
**Receiving information about lifestyle (Yes)**	235	72.8
**Need for additional information on** **healthy lifestyle**	135	41.8

* Mean ± standard deviation (range). ^a^ Number of daily cigarettes smoked by current smokers. ^b^ Numbers for each item may not add up to the total number of the study population due to missing values. **^c^** International Physical Activity Questionnaire.

**Table 3:** Multivariate logistic regression models to identify predictors of outcomes of interest regarding lifestyle habits (smoking and alcohol consumption, consumption of at least 5 daily portions of fruits and vegetables, and active physical activity).

Model 1. Having Never Smoked or Drunk Alcohol			
**Variable °**	**OR**	**95% CI**	***p***
Female	2.48	1.02–6.06	0.046
Married/cohabitant	2.43	1.17–5.04	0.017
Talking with students about alcohol consumption at school	2.35	1.07–5.12	0.033
Age in years (continuous)	1.03	0.99–1.06	0.184
Having a postgraduate degree (master, PhD)	3.78	0.84–17.07	0.083
Teaching humanistic disciplines	0.45	0.13–1.58	0.214
Teaching artistic disciplines	0.54	0.16–1.79	0.316
Teaching support disciplines	0.31	0.08–1.15	0.078
Being underweight/healthy weight	0.14	0.02–1.11	0.062
**Model 2. Consumption of at least 5 daily portions of fruits and vegetables**			
**Variable ^+^**	**OR**	**95% CI**	***p***
Age in years (continuous)	1.03	1.01–1.06	0.024
Female	2.24	1.11–4.49	0.024
Having a university degree	4.25	1.36–13.29	0.013
Having a postgraduate degree (master, PhD)	4.23	1.14–15.74	0.031
Being overweight	2.87	1.02–8.09	0.046
Being underweight/healthy weight	2.33	0.85–6.35	0.099
Minimally active/active/very active IPAQ score	2.14	1.31–3.55	0.003
Having healthy behavior regarding cigarette smoking and alcohol consumption	1.86	0.86–4.01	0.114
Talking with students about physical activity at school	0.72	0.44–1.19	0.207
**Model 3. Active physical activity assessed using IPAQ score**			
**Variable ^§^**	**OR**	**95% IC**	***p***
Having at least one child	0.52	0.29–0.93	0.027
Teaching humanistic disciplines	0.53	0.29–0.94	0.029
Teaching support disciplines	0.33	0.16–0.71	0.004
Consumption of at least 5 daily portions of fruits and vegetables	2.38	1.43–3.95	0.001
Needing additional information on healthy lifestyle habits	0.55	0.34–0.91	0.019
Female	0.74	0.38–1.42	0.359
Talking with students about physical activity at school	1.49	0.91–2.15	0.121

° The following variables were deleted by the backward elimination procedure: talking with students about cigarette smoking at school, having a university degree, and being overweight. ^+^ The following variable was deleted by the backward elimination procedure: children. **^§^** The following variables were deleted by the backward elimination procedure: age in years (continuous), marital status, receiving information about lifestyle habits, and teaching artistic disciplines.

**Table 4:** Multivariate logistic regression models used to identify predictors of having discussed at least one topic among nutrition, physical activity, cigarette smoking, and alcohol consumption with students at school.

Model 4. Having Discussed at Least One Topic Among Nutrition, Physical Activity, Cigarette Smoking, and Alcohol Consumption With Students at School			
Variable ^@^	OR	95% CI	*p*
Female	0.39	0.19–0.78	0.007
Having a university degree	4.55	1.64–12.64	0.004
Having a postgraduate degree (master, PhD)	2.16	0.65–7.20	0.209
Teaching humanistic disciplines	0.43	0.21–0.89	0.022
Teaching artistic disciplines	0.44	0.22–0.88	0.02
Teaching support disciplines	0.38	0.16–0.91	0.03
Age in years (continuous)	1.02	0.99–1.04	0.247
Being underweight/healthy weight	0.81	0.48–1.32	0.39

^@^ The following variables were deleted by the backward elimination procedure: marital status, being overweight.

**Table 5:** Teachers’ correct knowledge regarding screening activities.

Knowledge	N	%
Mammography is free of charge for women aged 45–69 years *^a^	220	84.2
Mammography is recommended at two-year intervals *^a^	121	46.2
Pap test is free of charge for women aged 25–64 years *^a^	147	57.9
Pap test is recommended at three-year intervals *^a^	61	23.5
HPV-DNA test is free of charge for women aged 30–64 years *^a^	128	51.2
HPV-DNA test is recommended at five-year intervals *^a^	39	16.5
FOBT is free of charge for women aged 50–69 years ^a^	223	69.1
FOBT is recommended at two-year intervals *^a^	129	39.9

* Only among women participants. ^a^ Numbers for each item may not add up to the total number of the study population due to missing values.

**Table 6:** Participation in oncological screening activities among age-eligible participants.

Oncological Screening Activity	Eligible	Never Performed/Do Not Remember	Performed at Least Once	Performed as Diagnostic Exam	Screening Test			Adherence to Screening Interval ^#^	
					Total	Opportunistic	Organized	Correct	Incorrect
	Total	N (%) *	N (%) *	N (%) *	N (%) *	N (%) *	N (%) *	N (%)	N (%)
**Mammography ^a^**	208	18 (8.7)	188 (91.3)	88 (50)	88 (50)	39 (44.3)	49 (55.7)	163 (90.6)	17 (9.4)
**Pap test ^b^**	246	12 (4.9)	231 (95.1)	143(62.5)	86 (37.5)	59 (68.6)	27 (31.4)	191 (83.1)	39 (16.9)
**HPV-DNA test ^c^**	244	161 (67.9)	76 (32.1)	38 (54.3)	32 (45.7)	15 (46.9)	17 (53.1)	77 (100)	-
**FOBT ^d^**	208	130 (62.5)	78 (37.5)	29 (40.3)	43 (59.7)	9 (20.9)	34 (79.1)	47 (62.7)	28 (37.3)

* Numbers for each item may not add up to the total number of the study population due to missing values. ^a^ Only among women age-eligible for mammography (aged 45–69 years). ^b^ Only among women age-eligible for the Pap test (aged 25–64 years). ^c^ Only among women age-eligible for the HPV-DNA test (aged 30–64 years). ^d^ Only among those age-eligible for the FOBT (aged 50–69 years). ^#^ Adherence to the correct screening interval: two years for mammography and the FOBT, three years for the Pap test, and five years for the HPV-DNA test.

**Table 7:** Multivariate logistic regression models to identify predictors of uptake in the different screening programs.

Model 5. Mammography Uptake in a Screening Program			
**Variable °**	**OR**	**95% CI**	***p***
Having a university degree	0.32	0.11–0.97	0.046
Having a postgraduate degree (master, PhD)	0.49	0.13–1.82	0.286
Receiving information about screening programs	3.56	1.39–9.11	0.008
Number of cohabitants (continuous)	1.19	0.92–1.52	0.182
Having at least one family member with a non-communicable disease	3.78	0.84–17.07	0.083
Teaching in a primary school	1.73	0.61–4.92	0.305
**Model 6. Pap test uptake in a screening program**			
**Variable ^+^**	**OR**	**95% CI**	***p***
Being married/cohabitant	2.59	1.22–5.47	0.013
Having at least one family member with a non-communicable disease	2.11	1.13–4.33	0.021
Having a university degree	0.31	0.11–0.87	0.026
Having a postgraduate degree (master, PhD)	0.45	1.13–1.57	0.212
Knowing that the Pap test is free of charge for women aged 25–64 years	2.11	1.22–5.47	0.013
Receiving information about screening programs	2.29	0.95–5.55	0.065
**Model 7.** **FOBT uptake in a screening program**			
**Variable**	**OR**	**95% IC**	***p***
Age in years (continuous)	1.09	1.02–1.18	0.014
Female	1.77	0.55–5.71	0.338
Having at least one child	1.76	0.65–4.78	0.265
Having at least one family member with a non-communicable disease	1.79	0.82–3.89	0.141
Having healthy behavior regarding cigarette smoking and alcohol consumption	4.06	0.51–32.57	0.187
Consumption of at least 5 daily portions of fruits and vegetables	1.43	0.69–2.96	0.334
Receiving information about screening programs	3.91	0.85–17.97	0.080

° The following variables were deleted by the backward elimination procedure: having healthy behavior regarding cigarette smoking and alcohol consumption, teaching in a middle school, and teaching in a high school. ^+^ The following variables were deleted by the backward elimination procedure: age in years (continuous), BMI category, and children.

## Data Availability

The data that support the findings of this study are available from the corresponding author upon reasonable request, to ensure participants’ privacy and anonymity.

## Supplementary Materials

The following supporting information can be downloaded at https://www.mdpi.com/article/10.3390/healthcare13233080/s1: Supplementary File S1: Questionnaire; Supplementary File S2: Independent variables included in the different final models.

## Abbreviations

The following abbreviations are used in this manuscript:

PA	Physical activity
BMI	Body Mass Index
Pap test	Papanicolaou test
HPV DNA test	Human papillomavirus DNA test
FOBT	Fecal occult blood test
IPAQ-SF	International Physical Activity Questionnaire—Short Form
MET	Metabolic Equivalent of Task
PSQI	Pittsburgh Sleep Quality Index
OR	Odds ratio
CI	Confidence interval
