# Supplementary material for: Lifestyle Habits and Adherence to Cancer Screening Programs Among Italian Teachers: A Cross-Sectional Study
Source: Healthcare (Basel). 2025 Nov 26;13(23):3080. doi: 10.3390/healthcare13233080 (PMC12692436; doi:10.3390/healthcare13233080)
Supplement: Supplementary file 1 [file healthcare-13-03080-s001.zip › Supplementary File S1.pdf]

QUESTIONNAIRE

A. SOCIO-DEMOGRAPHIC, PROFESSIONAL, AND ANAMNESTIC, CHARACTERISTICS

This section is designed to gather information about your socio-demographic, professional, anamnestic, and health-related characteristics.

A1. How old were you on your last birthday? ____________ **A2.** What is your gender? □ Female □ Male

A3. What is your marital status? □ Married □ Cohabiting □ Single (never married) □ Other (*please specifiy*) ____________________

A4. What is your highest educational level? □ High school □ University □ Post degree □ Other (*please specifiy)*____________

A5. If you have a partner, what is your partner's highest educational level? □ None □ Primary school □ High school □ University degree □ Post degree □ Other (*please specify)*___________________ (if you do not have not a partner, go to **A6**)

A6. In which type of school do you teach? □ Kindergarden □ Primary school □ High school □ Middle school

A7. What is the main subject you teach at school? ___________________

A8. If you have a partner, what is his/her job? (if you do not have a partner, go to **A9**) ________________

A9. Do you have any non-communicable disease? □ No □ Yes (*please specify*, more than one disease is allowed)

Disease	How long time?

A10. How many people live with you? *(indicate the number, excluding yourself)* _____

A11. Do you have children? □ No □ Yes, please specify how many _____

A12. Do your cohabitants have any non-communicable disease? □ No □ Yes (please specify age and disease, more than one disease is allowed)

Cohabitant age	Disease

A13. Indicate your weight: __ Kg **A14.** Indicate your height: __ cm

B. LIFESTYLE BEHAVIORS

The questions in this section aim to acquire information on your lifestyle behaviors.

**B1.** In the last 12 months, has you had a follow-up visit from the following health professionals?		
General practitioner	□ Yes □ No □ I don't remember	If yes, indicate how many times ____ and the reason ________________
Oculist	□ Yes □ No □ I don't remember	If yes, indicate how many times ____ and the reason ________________
Dentist	□ Yes □ No □ I don't remember	If yes, indicate how many times ____ and the reason ________________
Dermatologist	□ Yes □ No □ I don't remember	If yes, indicate how many times ____ and the reason ________________
Psychologist	□ Yes □ No □ I don't remember	If yes, indicate how many times ____ and the reason ________________
Other, please specify _______	□ Yes □ No □ I don't remember	If yes, indicate how many times ____ and the reason ________________

B2. Have you ever smoked cigarettes in your life?

□ No (go to **B4**) □ Yes

- If yes, do you currently smoke? □ No □ Yes;
- If yes, how many cigarettes do you smoke per day? (indicate **number)** ____________
- If yes, what kind of cigarettes do you smoke?
  □ Traditional cigarettes □ Tobacco □ IQOS □ Vape cigarettes with nicotine
  □ Vape cigarettes without nicotine □ Other, (*please specify)*___________________

B3. At what age did you start smoking? (indicate **age**) ______________

B4. During the last 7 days, how many days did people around you smoke at home, at school, or in other environments that you frequented? □ 0 □ from 1 to 2 □ from 3 to 4 □ from 5 to 6 □ 7

B5. How often do you consume alcoholic drink (wine, beer, etc.)?

□ 1 time per month or less □ 2 to 4 times per month □ 2 or 3 times per week □ 4 or more times per week

B6. On the days you drink, how many alcoholic beverages do you consume on average?

□ 1 or 2 □ 3 or 4 □ 5 or 6 □ 7 or 9 □ 10 or more

B7. On which days of the week do you usually have breakfast? (*please specify*, more than one disease is allowed)

□ I never have breakfast □ Monday □ Tuesday □ Wednesday □ Thursday □ Friday □ Saturday □ Sunday

B8. On a typical day, what do you eat for breakfast? ____

B9. How many times a week do you consume the following products? *(one answer per line)*

	**Never**	**Less than once a week**	**Once a week**	**2/4 days per week**	**5/6 days a week**	**Once a day, every day**	**More than once a day**
**Fruit**	□	□	□	□	□	□	□
**Vegetables**	□	□	□	□	□	□	□
**Dairy products** (milk, cheese, yogurt, cream, etc.)	□	□	□	□	□	□	□
**Bread**	□	□	□	□	□	□	□
**Pasta**	□	□	□	□	□	□	□
**Pizza** (mini pizzas, etc.)	□	□	□	□	□	□	□
**Legumes** (lentils, beans, etc.)	□	□	□	□	□	□	□
**Eggs**	□	□	□	□	□	□	□
**Meat**	□	□	□	□	□	□	□
**Fish**	□	□	□	□	□	□	□
**Sweets** (snacks, cakes, chocolate, etc.)	□	□	□	□	□	□	□
**Fried foods** (chips, croquettes, etc.)	□	□	□	□	□	□	□
**Sugary drinks** (coca cola, fruit juices, etc.)	□	□	□	□	□	□	□
**Caffeinated drinks** (coffee, energy drink, tea, etc.)	□	□	□	□	□	□	□
**Sauces** (ketchup, mayonnaise, mustard, etc.)	□	□	□	□	□	□	□

The following questions will ask you about the time you spent being physically active in the last 7 days. Please answer each question, even if you do not consider yourself to be an active person. Please think about the activities you do at work, as part of your house and yard work, to get from place to place, and in your spare time for recreation, exercise or sport. Think about all the vigorous and moderate activities that you did in the last 7 days. Vigorous physical activities refer to activities that take hard physical effort and make you breathe much harder than normal. Moderate activities refer to activities that take moderate physical effort and make you breathe somewhat harder than normal.

B10. During the last 7 days, on how many days did you do vigorous physical activities like heavy lifting, digging, heavy construction, or climbing upstairs as part of your work? Think about only those physical activities that you did for at least 10 minutes at a time.

□ 1 □ 2 □ 3 □ 4 □ 5 □ 6 □ 7 □ No vigorous physical activity (go to **B12**)

B11. How much time did you usually spend on one of those days doing vigorous physical activities as part of your work? N°_____ of minutes per day

Now think of all those moderate activities you have done in the last seven days for at least 10 minutes straight. For moderate activities, we mean those that require a moderate physical effort and that make breathing with a rhythm a little more frequent than normal.

B12. Again, think about only those physical activities that you did for at least 10 minutes at a time. During the last 7 days, on how many days did you do moderate physical activities like carrying light loads as part of your work? Please do not include walking.

□ 1 □ 2 □ 3 □ 4 □ 5 □ 6 □ 7 □ No moderate job-related physical activity (go to **B14)**

B13. How much time did you usually spend on one of those days doing moderate physical activities as part of your work?

N° ___of minutes per day

Now think of the time that you spent walking in the last seven days. Include time spent in school, at home, generally moving from one place to another, and any other walks done for fun, sport, exercise, or pastimes.

B14. During the last 7 days, on how many days did you walk for at least 10 minutes at a time as part of your work? Please do not count any walking you did to travel to or from work.

□ 1 □ 2 □ 3 □ 4 □ 5 □ 6 □ 7 □ No job-related walking (go to **B16)**

B15. How much time did you usually spend on one of those days walking as part of your work? N° _______ minutes per day (for example, it can be 30 minutes to go to work, 15 minutes to get to transport, 15 minutes to walk the dog)

The last questions are about the time you spend sitting while at work, at home, while doing course work and during leisure time. This may include time spent sitting at a desk, visiting friends, reading or sitting or lying down to watch television. Do not include any time spent sitting in a motor vehicle that you have already told me about.

B16. During the last seven days, how much time did you usually spend sitting on a day? Consider the time at the desk, reading, lying/watching television) N° ________ of minutes per day

The following questions relate to your usual sleep habits during the past month only. Your answers should indicate the most accurate reply for the majority of days and nights in the past month. Please answer all questions. During the past month…

B17. When have you usually gone to bed? _______

B18. How long (in minutes) has it taken you to fall asleep each night? _____________

B19. When have you usually gotten up in the morning? ________

B20. How many hours of actual sleep do you get at night? (*This may be different than the number of hours you spend in bed*) ___________

**B21**. During the past month, how often have you had trouble sleeping because you…	Not during the past month (0)	Less than once a week (1)	Once or twice a week (2)	Three or more times week (3)
**B21a.** Cannot get to sleep within 30 minutes				
**B21b.** Wake up in the middle of the night or early morning				
**B21c.** Have to get up to use the bathroom				
**B21d.** Cannot breathe comfortably				
**B21e.** Cough or snore loudly				
**B21f.** Feel too cold				
**B21g.** Feel too hot				
**B21h.** Have bad dreams				
**B21i.** Have pain				
**B21j.** Other reason(s), please describe, including how often you have had trouble sleeping because of this reason(s):				
**B22.** During the past month, how often have you taken medicine (prescribed or "over the counter") to help you sleep?				
**B23.** During the past month, how often have you had trouble staying awake while driving, eating meals, or engaging in social activity?				
**B24.** During the past month, how much of a problem has it been for you to keep up enthusiasm to get things done?				
	Very good (0)	Fairly good (1)	Fairly bad (2)	Very bad (3)
**B25.** During the past month, how would you rate your sleep quality overall?				

INFORMATION

B26. On which of the following lifestyle behaviors did you receive information?

□ Smoking □ Alcohol □ Nutrition □ Physical activity □ Sleep

B27. What are your sources of information about lifestyle behaviors? (more than one source is allowed)?

□ None □ Physicians □ Mass media □ Internet □ Family members/Friends □ School/University □ Other (*please specify)* ______________________

B28. Do you feel you need more information about the correct lifestyle behaviors? □ No □ Yes

B29. Which of the following topics did you discuss with your students in class? (more than one topic is allowed)

□ None □ Cigarette smoking □ Alcohol □ Nutrition □ Physical activity

C. KNOWLEDGE AND PARTICIPATION IN SCREENING PROGRAMMES (Males need only answer questions from C13 to C18).

C1. Mammography uses X-ray to detect breast cancer and is recommended for women from 45 to 69 years old every 2 years. Who is it offered free of charge?
□ To all women □ To women between 25 and 44 years old
□ To women between 45 and 69 □ To women over 69
C2. How often should a mammogram be done?
□ Every year □ Every 2 years □ Every 5 years
C3. Have you ever done it?
□ No, why? (even more than one response)
□ I have not received recommendation by physician
□ I do not have any health problem
□ Lack of time
□ Too long waiting list
□ I am afraid of discovering the disease
□ I am afraid of pain
Other (please specify) __________
□ I don't remember (go to **C5**)
□ Yes, why? (even more than one response)
□ For signs and symptoms, (please specify) __________
□ For control □ Voluntarily (eg. Private medical examination)
□ Screening campaign
C4. When? □ In the last year □ 2 years ago □ 3 years ago
□ 4 years ago □ ≥ 5 years ago

C5. Papanicolaou test (PAP-test) collects cells from uterus to detect cervical cancer and is recommended for women aged 25-64 every 3 years. Who is it offered free of charge?
□ To all women □ To women between 25 and 64
□ To women over 64
C6. How often should the Pap test be performed?
□ Every year □ Every 3 years □ Every 5 years
C7. Have you ever done it?
□ No, why? (even more than one response)
□ I have not received recommendation by physician
□ I do not have any health problem
□ Lack of time
□ Too long waiting list
□ I am afraid of discovering the disease
□ I am afraid of pain
Other (please specify) __________
□ I don't remember (go to **D9**)
□ Yes, why? (even more than one response)
□ For signs and symptoms, (please specify) __________
□ For control □ Voluntarily (eg Private medical examination)
□ Screening campaign
C8. When? □ In the last year □ 2 years ago □ 3 years ago
□ 4 years ago □ ≥ 5 years ago

C9. HPV-DNA test collects cells from the uterus to detect the Papilloma Virus (HPV) and is recommended for women aged 30-35 to 64 every 5 years. Who is it offered free of charge?
□ To all women □ To women between 30 and 64
□ To women over 64
C10. How often should the HPV-DNA test be performed?
□ Every year □ Every 3 years□ Every 5 years □ Every 10 years
C11. Have you ever done it?
□ No, why? (even more than one response)
□ I have not received recommendation by physician
□ I do not have any health problem
□ Lack of time
□ Too long waiting list
□ I am afraid of discovering the disease
□ I am afraid of pain
Other (please specify) __________
□ I don't remember (go to **D13**)
□ Yes, why? (even more than one response)
□ For signs and symptoms, (please specify) __________
□ For control □ Voluntarily (eg. Private medical examination)
□ Screening campaign
C12. When? □ In the last year □ 2 years ago □ 3 years ago
□ 4 years ago □ ≥ 5 years ago

C13. The fecal occult blood test (FOBT) is used to determine the presence of blood in the stool. Who is it offered free of charge?
□ To all persons □ To persons between 50 and 69 years of age
□ To persons over 69 years of age
C14. How often should the FOBT test be performed?
□ Every year □ Every 2 years□ Every 5 years □ Every 10 years
C15. Have you ever done it?
□ No, why? (even more than one response)
□ I have not received recommendation by physician
□ I do not have any health problem
□ Lack of time
□ Too long waiting list
□ I am afraid of discovering the disease
□ I am afraid of pain
Other (please specify) __________
□ I don't remember (go to **D17**)
□ Yes, why? (even more than one response)
□ For signs and symptoms, (please specify) __________
□ For control □ Voluntarily (eg. Private medical examination)
□ Screening campaign
C16. When? □ In the last year □ 2 years ago □ 3 years ago
□ 4 years ago □ ≥ 5 years ago

INFORMATION
C17. What are your sources of information about screening programmes? (more than one source is allowed)?
□ None □ Physicians □ Mass media □Internet □ Family members/Friends □ School/University □ Other (*please specify)* __________
C18. Do you feel you need more information about screening programmes? □ No □ Yes
