# Supplementary material for: Lifestyle Habits and Adherence to Cancer Screening Programs Among Italian Teachers: A Cross-Sectional Study
Source: Healthcare (Basel). 2025 Nov 26;13(23):3080. doi: 10.3390/healthcare13233080 (PMC12692436; doi:10.3390/healthcare13233080)
Supplement: Supplementary file 1 [file healthcare-13-03080-s001.zip › Supplementary File S2.pdf]

Supplementary File S2

The independent variables included in the different final models.

Model	Outcome	Independent variables
**1**	Having never smoked and never drunk alcohol<br>(no=0; yes=1)	• age in years (continuous)<br>• gender (male=0; female=1)<br>• marital status (unmarried/widowed/divorced=0; married/cohabitant=1)<br>• education level (high school=1; university degree=2; postgraduate degree (master, PhD)=3)<br>• subjects (humanistic=1; science=2; artistic=3; support=4)<br>• BMI (underweight/healthy weight=1; overweight=2; obese=3)<br>• talking with students about cigarette smoking (no=0; yes=1)<br>• talking with students about alcohol consumption (no=0; yes=1)
**2**	Eating at least 5 daily portions of fruits and vegetables<br>(no=0; yes=1)	• age in years (continuous)<br>• gender (male=0; female=1)<br>• children (no=0; at least one=1)<br>• education level (high school=1; university degree=2; postgraduate degree (master, PhD)=3)<br>• BMI (underweight/healthy weight=1; overweight=2; obese=3)<br>• having healthy behavior regarding cigarette smoking and alcohol consumption (no=0; yes=1)<br>• talking with students about physical activity (no=0; yes=1)<br>• physical activity (inactive=0; minimally active/active/very active=1)
**3**	Having a moderate/high level of physical activity<br>(no=0; yes=1)	• age in years (continuous)<br>• gender (male=0; female=1)<br>• marital status (unmarried/widowed/divorced=0; married/cohabitant=1)<br>• children (no=0; at least one=1)<br>• subjects (humanistic=1; science=2; artistic=3; support=4)<br>• eating at least 5 daily portions of fruits and vegetables (no=0; yes=1)<br>• talking with students about physical activity (no=0; yes=1)<br>• receiving information about lifestyle habits (no=0; yes=1)<br>• needing additional information on healthy lifestyle habits (no=0; yes=1)

**4**	Having discussed at least one topic among nutrition, physical activity, cigarette smoking, and alcohol consumption with students at school<br>(no=0; yes=1)	• age in years (continuous)<br>• gender (male=0; female=1)<br>• marital status (unmarried/widowed/divorced=0; married/cohabitant=1)<br>• education level (high school=1; university degree=2; postgraduate degree (master, PhD)=3)<br>• subjects (humanistic=1; science=2; artistic=3; support=4)<br>• BMI (underweight/healthy weight=1; overweight=2; obese=3)
**5**	Having had a mammogram for breast cancer screening<br>(no=0; yes=1)	• number of cohabitants (continuous)<br>• family with non-communicable diseases<br>• education level (high school=1; university degree=2; postgraduate degree (master, PhD)=3)<br>• having healthy behavior regarding cigarette smoking and alcohol consumption (no=0; yes=1)<br>• receiving information about screening programs (no=0; yes=1)<br>• type of school (kindergarten=1; primary school=2; middle school=3; high school=4)
**6**	Having had a Pap smear for cervical cancer screening<br>(no=0; yes=1)	• age in years (continuous)<br>• marital status (unmarried/widowed/divorced=0; married/cohabitant=1)<br>• children (no=0; at least one=1)<br>• family with non-communicable diseases<br>• education level (high school=1; university degree=2; postgraduate degree (master, PhD)=3)<br>• BMI (underweight/healthy weight=1; overweight=2; obese=3)<br>• receiving information about screening programs (no=0; yes=1)<br>• knowledge that Pap test is free of charge for women aged 25-64 years (incorrect=0; correct=1)
**7**	Having had a FOBT for colorectal cancer screening<br>(no=0; yes=1)	• age in years (continuous)<br>• gender (male=0; female=1)<br>• children (no=0; at least one=1)<br>• family with non-communicable diseases<br>• having healthy behavior regarding cigarette smoking and alcohol consumption (no=0; yes=1)<br>• eating at least 5 daily portions of fruits and vegetables (no=0; yes=1)<br>• receiving information about screening programs (no=0; yes=1)
